# Beyond catalysis: Spatial topology of GPX4 in ferroptosis and its theranostic implications

**DOI:** 10.7150/thno.134666

**Published:** 2026-05-26

**Authors:** Fantian Zeng, Feida Li, Huifen Guo, Guoao Dong, Zijian Zhou, Xiaoyuan Chen

**Affiliations:** 1 State Key Laboratory of Vaccines for Infectious Diseases, Xiang’An Biomedicine Laboratory & Center for Molecular Imaging and Translational Medicine, National Innovation Platform for Industry-Education Integration in Vaccine Research, School of Public Health, Xiamen University, Xiamen 361102, China.; 2 Center for Nuclear Medicine and Molecular Imaging (CNMMI), Shandong Cancer Hospital and Institute, Jinan, 250117, China.

## Abstract

As the primary gatekeeper of ferroptosis, glutathione peroxidase 4 (GPX4) represents a compelling therapeutic target. A recent study demonstrates that sustained GPX4 blockade via sophisticated lipidic nanoplatforms simultaneously triggers robust ferroptosis and remodels the tumor immune microenvironment; however, the underlying synergistic mechanisms remain poorly elucidated. By integrating classical GPX4 enzymatic theories with recent breakthroughs regarding its membrane-bound structural functions and its secretion as an immune-disrupting ligand, we propose a framework illustrating how GPX4 serves as a nexus between intracellular survival mechanisms and extracellular immune evasion. More importantly, we delineate a "spatial topological" landscape of GPX4 to fully harness its therapeutic potential in cancer therapy.

Glutathione peroxidase 4 (GPX4) has long been recognized as the central gatekeeper of ferroptosis 1-3. Historically, translational strategies targeting GPX4 have primarily focused on inhibiting its catalytic activity. First-generation covalent inhibitors, including RSL3 and ML162, were developed to induce ferroptosis by targeting the selenocysteine residue within the enzyme’s active site. Although these agents are mechanistically sound at the biochemical level, their clinical advancement has been limited by unfavorable pharmacokinetics, off-target toxicities, and the emergence of tumor-cell adaptive resistance. Furthermore, it is now widely recognized that GPX4 does not operate in isolation. Parallel ferroptosis defense systems, such as the FSP1-CoQ10 axis 1 and the mitochondria-localized DHODH pathway 3, provide powerful compensatory mechanisms. These parallel networks explain why simply inhibiting the enzymatic activity of GPX4 is frequently insufficient to completely eradicate tumor cells

A key underlying limitation is that covalent inhibition strategies may have underestimated the spatiotemporal complexity and adaptive plasticity of GPX4 within cellular systems. GPX4 functions not merely as a soluble antioxidant enzyme but as a dynamically regulated protein whose activity and biological consequences depend on its subcellular localization and contextual interactions. Consequently, overcoming current pharmacologic barriers requires moving beyond a reductionist enzyme-centric framework toward a spatially informed perspective. Here, we propose a conceptual framework of “spatial topology”, which dissects how GPX4 executes multidimensional functions through distinct functional identities across the cytoplasm, the cell membrane, and the extracellular microenvironment.

## Intracellular: canonical enzymatic activity

In the intracellular compartment, accumulating evidence suggests that GPX4 function is governed by a highly dynamic proteostasis network and post-translational modifications (PTMs), rather than by simple substrate catalysis alone. Traditional inhibitors often fail because they require sufficiently high concentrations to continuously saturate the active site, which frequently leads to toxicity in sensitive tissues such as the kidneys 4. Moreover, tumor cells exhibit powerful adaptability by stabilizing GPX4 protein levels through complex PTM networks to resist therapeutic pressure. Beyond the established role of deubiquitinases like OTUB1 and USP7 5, recent research has unveiled the core status of S-palmitoylation in GPX4 homeostasis. The acyltransferase ZDHHC20 attaches a palmitate moiety to cysteine 66, a modification that not only stabilizes GPX4 at cellular membranes but also protects it from degradation machinery 6. Conversely, the depalmitoylase APT2 removes this lipid modification, sensitizing cells to ferroptosis. This "palmitoylation-depalmitoylation" cycle acts as a rapid homeostatic switch, explaining why tumors with active lipid metabolism often possess innate resistance. Furthermore, phosphorylation adds another layer of defense. In hepatocellular carcinoma, Secernin-1 (SCRN1) scaffolds the kinase STK38 to phosphorylate GPX4 at serine 45 7, thereby structurally stabilizing the protein and compromising the efficacy of conventional enzymatic inhibitors.

## Membrane-bound: structural stability

At the cell membrane, recent studies have addressed a long-standing biophysical paradox: how can GPX4, a predominantly soluble protein rich in hydrophilic amino acids, effectively access the hydrophobic core of the phospholipid bilayer to reduce peroxidized lipid acyl chains buried deep within the membrane? High-resolution structural analysis has identified a previously overlooked hydrophobic loop on the GPX4 surface 8, termed the “fin-loop”. This unique structure functions much like a shark fin, enabling GPX4 to dynamically embed itself within the cell membrane. This insertion is not simply the result of random thermal motion but represents a finely tuned structural adaptation. Once anchored to the membrane via its fin loop, GPX4 functions not merely as a conventional enzyme acting near its substrates, but also as a critical structural scaffold. By physically occupying space within the lipid leaflet, GPX4 blocks the wave-like propagation of lipid peroxidation chain reactions across the membrane plane. This physical barrier effect is particularly vital in neuronal membranes rich in polyunsaturated fatty acids (PUFAs). Clinical genetic evidence strongly supports this paradigm. Patients harboring the GPX4 R152H mutation develop early-onset neurodegenerative diseases, and the mutation localizes precisely to the fin-loop region.

Moreover, GPX4 membrane localization is not a spontaneous process; it requires directed recruitment by the molecular chaperone peroxiredoxin 6 (PRDX6) 9. PRDX6 escorts cytosolic GPX4 to calcium- and lipid-rich membrane microdomains such as lipid rafts. In malignant tumors such as glioblastoma, the PRDX6–GPX4 axis is frequently hyperactivated. In addition, lipid droplets—intracellular organelles for lipid storage—have been shown to participate in a finely tuned interplay with GPX4’s membrane-associated activity. As reported by Chauhan *et al.*, hypoxia-induced prostate cancer cells alleviate membrane oxidative stress by enhancing lipid droplet biogenesis, which sequesters oxidation-prone PUFAs and thereby relieves the detoxification burden on membrane-bound GPX4 10. This underscores a coordinated cell-survival mechanism linking membrane lipid remodeling with spatial regulation of GPX4.

## Extracellular: immune evasion

In the extracellular space, recent studies have shown that GPX4 is not confined to the cell membrane; tumor cells actively release it into the microenvironment through exosomes or non-canonical secretion pathways. This observation aligns with the broader concept of the tumor as an ecosystem, wherein exosomes act as critical signaling hubs that transport diverse molecular cargo to remodel the metabolic and immune landscapes. While exosomes are well known for delivering metabolic regulators and miRNAs that promote tumor growth or prime the pre-metastatic niche, the secretion of GPX4 represents a distinct mechanism of immune evasion. Once released into the extracellular space, GPX4 undergoes a striking functional transition, shifting from an intracellular antioxidant enzyme to an immunosuppressive ligand.

Further studies have shown that extracellular GPX4 specifically binds to the zona pellucida glycoprotein 3 (ZP3) receptors on the surface of dendritic cells (DCs) 11. This GPX4-ZP3 interaction initiates inhibitory signaling cascades within DCs, substantially impairing their maturation, as well as their antigen processing and presentation capacities. Ultimately, this results in the exhaustion and dysfunction of downstream CD8⁺ T cells. Through this mechanism, tumor cells effectively cultivate an immunosuppressive “cold” microenvironment. Rather than acting merely as a byproduct of cell stress, secreted GPX4 participates in a specific immunosuppressive pathway. While further studies are required to determine if it functions as a canonical immune checkpoint comparable to PD-L1, it undeniably serves as a critical mediator of immune evasion. Even when experiencing internal oxidative stress, tumor cells exploit secreted GPX4 as a specific damage-associated molecular pattern (DAMP) modulator, transmitting a deceptive “do not attack” signal to surrounding immune cells. Thus, GPX4 embodies a “double-edged sword” regarding the fate of the immune cells themselves. A comprehensive summary of these multidimensional spatial regulatory mechanisms is provided in Table 1.

To effectively overcome this therapeutic paradox, next-generation interventions need to simultaneously dismantle the intracellular GPX4 defense networks and neutralize its extracellular immunosuppressive role. A recent study demonstrates the feasibility of this dual spatial blockade 12. By employing a ligand-directed lipidic nanoplatform for metronomic delivery, this approach continuously overrides intracellular compensatory mechanisms to drive sustained ferroptosis. In this study, the authors experimentally observed significant immune remodeling, characterized by an increased infiltration of antigen-presenting cells and M1 macrophages, alongside a reduction in immunosuppressive regulatory T cells. While the exact mechanistic link remains to be fully elucidated, we infer that dismantling the tumor cell architecture via robust ferroptosis may effectively cut off the source of secreted GPX4, potentially alleviating the GPX4-ZP3-mediated immune suppression.

## Dynamic crosstalk and context-dependent topology

Crucially, the intracellular, membrane-associated, and extracellular compartments of GPX4 do not function in isolation; rather, they are linked through continuous and regulated dynamic trafficking. The subcellular prioritization of GPX4 is highly context-dependent. Under normal physiological homeostasis, the spatial topology of GPX4 is tightly regulated to maintain tissue integrity. For instance, in developing spermatozoa, GPX4 acts as an essential structural protein in the mitochondrial capsule 13, whereas in mature neurons, its membrane-anchored form is vital for preventing lipid peroxidation in PUFA-rich neural networks. However, in malignant microenvironments, tumor cells hijack this dynamic trafficking. Stress-induced signals promote the rapid translocation of cytosolic GPX4 to the plasma membrane via PRDX6 chaperoning, while simultaneously hyperactivating non-canonical secretion pathways to release GPX4 into the extracellular matrix. Understanding this pathological spatial hijacking is critical for developing therapies that disrupt tumor-specific GPX4 trafficking without causing fatal toxicity to normal tissues.

For the field of theranostics, elucidating this multidimensional spatial topology offers a promising roadmap for next-generation drug development. Future therapeutic strategies may increasingly focus on spatially selective interventions that target specific GPX4 pools within distinct cellular and extracellular compartments. Intracellular targeting could be enhanced by Targeted Protein Degradation (TPD) technologies, such as Proteolysis Targeting Chimeras (PROTACs) like dGPX4 14, or cell-penetrating nanobodies, like camelid-derived 12E 15. These approaches physically direct GPX4 to the proteasome for degradation, effectively bypassing resistance mechanisms. Targeting the membrane-associated fraction of GPX4 presents a distinct therapeutic strategy. Small molecules that block fin-loop insertion or disrupt the PRDX6–GPX4 interaction could selectively compromise the structural barrier of tumor cells, sensitizing them to treatment while sparing basal cytosolic metabolism. Furthermore, addressing the extracellular compartment presents an opportunity to navigate the complex role of GPX4 in immune cells. Developing GPX4-specific neutralizing antibodies could selectively block the GPX4-ZP3 immune evasion axis without crossing the plasma membrane, thereby preserving the intracellular GPX4 activity required for the persistence and cytotoxic function of effector T cells. While these spatially defined interventions offer profound mechanistic advantages, they concurrently present distinct challenges (Table 2). By decoding this complex spatial regulatory network, the modulation of ferroptosis has the potential to evolve from broad enzymatic inhibition into a precise, multi-layered approach for cancer therapy.

## Figures and Tables

**Table 1:** Spatial regulatory mechanisms of GPX4 in tumor microenvironments

Spatial Compartment	Primary Function	Key Mediators	Regulatory Modifications	Functional Priority in Tumors
Intracellular (Cytosol/Mitochondria)	Substrate catalysis (Reduction of PL-OOH)	OTUB1, USP7, STK38	Deubiquitination Phosphorylation (Ser45)	Protein stability; Resistance to enzymatic inhibitors
Cell Membrane	Physical barrier against peroxidation chain reactions	PRDX6, ZDHHC20/APT2	S-palmitoylation (Cys66)	Membrane integrity maintenance under high metabolic stress
Extracellular	Immune evasion signal	ZP3 receptors	Exosomal packaging	Suppression of DC maturation and subsequent T-cell exhaustion

**Table 2:** Comparison of spatially targeted GPX4 therapeutic strategies

Targeting Strategy	Target Compartment	Mechanism of Action	Main Advantages	Current Limitations
Conventional Enzymatic Inhibitors (e.g., RSL3)	Intracellular	Covalent binding to active site	Well-established biochemical mechanisms	Off-target toxicity (e.g., renal failure); Rapid tumor resistance
Targeted Protein Degradation (PROTACs/Nanobodies)	Intracellular	Directs GPX4 protein to the proteasome for degradation	Bypasses PTM-mediated resistance; Completely removes the protein	Intracellular delivery efficiency; Large molecular weight
Fin-loop Blockers/Trafficking Inhibitors	Membrane- bound	Blocks GPX4 insertion or disrupts PRDX6-GPX4 interaction	Selectively collapses the tumor's physical lipid barrier	Requires precise structural drug design; Early stage of development
Neutralizing Antibodies	Extracellular	Blocks the GPX4-ZP3 interaction on immune cells	Reverses immune suppression while sparing intracellular GPX4	Cannot induce tumor ferroptosis alone; Requires combination therapy
